# Are recommended dietary patterns equitable?

**DOI:** 10.1017/S1368980021004158

**Published:** 2022-02

**Authors:** Vivian Hsing-Chun Wang, Victoria Foster, Stella S Yi

**Affiliations:** 1 Department of Public Health Policy and Management, School of Global Public Health, New York University, 708 Broadway, New York, NY 10003, USA; 2 Department of Population Health, NYU Grossman School of Medicine, New York, NY, USA

**Keywords:** Cultural adaptation, Racial/ethnic minority groups, Mediterranean diet, Dietary approaches to stop hypertension, EAT-Lancet, NOVA

## Abstract

**Objective:**

Dietary recommendations (DR) in the USA may be inadequate at improving diets in racial/ethnic minority communities and may require redesign of the systems driving their development over the long term. Meanwhile, cultural adaptation of evidence-based DR may be an important strategy for mitigating nutrition disparities, but less is known about the adaptability of these recommendations to meet the needs of diverse groups. We examined the content and origin of major DRs – aspects that provide context on their potential universality across populations and evaluated their potential for cultural adaptation.

**Design:**

Case studies of Dietary Approaches to Stop Hypertension (DASH), the Mediterranean diet (MD), the EAT-Lancet diet (EAT) and the NOVA classification system.

**Setting:**

United States.

**Participants:**

Racial/ethnic minority populations.

**Results:**

Current DR differ in their origin/evolution but are similar in their reductionist emphasis on physical health. DASH has been successfully adapted for some cultures but may be challenged by the need for intensive resources; MD may be more beneficial if applied as part of a broader set of food procurement/preparation practices than as just diet alone; EAT-Lancet adaptation may not honor existing country-specific practices that are already beneficial to human and environmental health (e.g. traditional/plant-based diets); evidence for cultural adaptation is limited with NOVA, but classification of levels of food processing has potential for widespread application.

**Conclusions:**

For DR to equitably support diverse populations, they must move beyond a Eurocentric or ‘general population’ framing, be more inclusive of cultural differences and honour social practices to improve diet and reduce disparities.

Adherence to dietary recommendations (DR) remains a challenge for the majority of Americans for a number of reasons including taste preferences, limitations in nutrition education or food preparation knowledge, or most importantly, the systemic barriers leading to inequitable access to healthy food. It is well established that racial/ethnic minorities and those at lower income levels in the USA have poorer diet quality compared with whites and individuals at higher income levels^(1,2)^. One dimension that has been less explored is the potential limitation of existing DR in considering diverse cultural values around food that may consequently compromise nutritional intake. Increasingly, immigrant communities – a large proportion which are Latina/x/o and Asian in the USA – maintain culinary traditions that are diverse and differ widely from a typical American dietary pattern^(3–7)^. The cultural dimension of eating, which is crucial for staying connected with cultural identity and community^(8)^, has rarely been accounted for related public health guidance, including the Dietary Guidelines for Americans (DGA)^(9–11)^.

Cultural adaptation can potentially bridge the gap between existing DRs and health equity^(12)^. Additionally, we put forth the notion that some recommendations may be more readily adapted to different cultures than others without significant increase in financial burden. With a focus on the diverse population in the USA, this commentary first briefly describes salient features of the DGA, then against this backdrop describes the specific content, origin, purpose and level of adaptability from prior cultural adaptations of the Dietary Approaches to Stop Hypertension, the Mediterranean Diet, the EAT-Lancet diet and the NOVA classification system. Similar to DGA, these widely used and emerging DR (i.e. patterns and frameworks) in the USA focus on physical well-being and lack attention to the cultural perspective that contributes to social and emotional health^(13–15)^. We conclude with suggestions for broadening the scope of cultural adaptation towards sustainable behavioural changes for nutritional health and general well-being among the racial/ethnic minority populations.

## Dietary guidelines for Americans

An overview of DGA is included in Table 1. While the DGA suggest considerations of ‘personal preferences, cultural traditions, and budgetary conditions’^(16)^, the recommendations are based on the intake of the general US population – data which underrepresent the preferences of diverse racial/ethnic minority subgroups^(17)^. Further, the DGA emphasise foods based on their nutrient density for the benefit of reducing disease risks^(16)^ – which runs counter to foodways of other cultures that prioritises social connections^(8)^. In other words, compliance to DGA means (1) prioritising physical health over social and emotional health and (2) adhering to a dietary pattern that does not account for cultural dimension, and therefore disproportionately impacts minorities in the USA. Lastly, the DGA inform federally funded food and nutrition programmes that disproportionately serve racial/ethnic minorities^(18,19)^, yet the mismatch of eating behaviours and preferences and reductionist nutrition in the absence of sociocultural influences may be inadequate to improve nutrition in these groups.

**Table 1:** Purpose, specific content, and origin and evolution of popular and emerging dietary recommendations in the United States

Name and purpose	Overall guideline and details	Origin and evolution
Dietary Guidelines for Americans^(16)^ To fulfill public health mission of promoting health, prolonging life, and preventing diet-related diseases such as obesity and diabetes at every life stage	Guidelines: (1) ‘follow a healthy dietary pattern at every life stage’; (2) ‘customize and enjoy nutrient-dense food and beverage choices to reflect personal preferences, cultural traditions, and budgetary considerations’; (3) ‘focus on meeting food group needs with nutrient-dense foods and beverages, and stay within calorie limits’ and (4) ‘limit foods and beverages higher in added sugars, saturated fat, and sodium, and limit alcoholic beverages.’^(16)^ Details: • dietary pattern by calorie (2000 kcal/d)—85 % daily calories to be filled with nutrient-dense food choices from six food groups; 15 % are discretionary calories such as added sugars and saturated fat^(16)^ • dietary pattern by food group (2000 kcal; equivalence/d): vegetables of varied colours and parts (2·5 cups), fruits (2 cups), grains (6 oz), dairy (3 cups), protein foods (5½ oz), oils (27 g), calories for other use (240 kcal)^(16)^	Origin • Updated every 5 years since 1992 as mandated by the Congress under the National Nutrition Monitoring and Related Research Act of 1990^(16)^ • A collaboration between United States Department of Agriculture and Department of Health and Human Services^(16)^ Evolution • For the first time, the ninth and latest version incorporates recommendations for women who are pregnant and infants/toddlers from birth to 24 months^(16)^ • For more details, refer to the DGA^(16)^
DASH^(50,51)^ To lower blood pressure	Guideline: (1) ‘eat vegetables, fruits, and whole grains; (2) ‘include fat-free or low-fat dairy products, fish, poultry, beans, nuts, and vegetable oils’; (3) ‘limit foods that are high in saturated fat, such as fatty meats, full-fat dairy products, and tropical oils such as coconut, palm kernel, and palm oils’; (4) ‘limit sugar-sweetened beverages and sweets’ and (5) ‘choose foods that are low in saturated fats and trans fats and rich in potassium, calcium, magnesium, fiber, and protein’^(50,51)^ Details: Dietary pattern by food group (servings/d unless indicated) – grains (6–8); meats, poultry and fish (≤ 6); vegetables (4–5); fruit (4–5); low-fat or fat-free dairy products (2–3); fats and oils (2–3); Na (2300 mg); nuts, seeds, dry beans and peas (4–5 servings/week) and sweets (≤ 5 servings/week)^(50,51)^	Origin • Rooted in a national health priority to treat hypertension; about 60 % of the participants in this original study were Black Americans to address the high prevalence of hypertension in this population at the time^(52)^ • Diets high in fibre and minerals such as potassium and Mg and low in fat are associated with low blood pressure • Recognised that adding nutrients alone may overlook other components of a food and bioavailability Evolution • Five key studies with varying Na levels (2300–3000 mg) suggested benefits to blood pressure control including lower LDL: DASH, DASH-sodium, Omniheart, OmniCarb and PREMIER trials^(13,51,53–55)^
Mediterranean Diet^(14)^ To promote cardiovascular health	Characteristics: (1) ‘abundance of plant foods (fruit, vegetables, breads, other forms of cereals, potatoes, beans, nuts, and seeds)’; (2) ‘minimally processed, seasonally fresh, and locally grown foods’; (3) ‘fresh fruit as the typical daily dessert, with sweets containing concentrated sugars or honey a few times/week’; (4) ‘olive oil as the principal source of fat’; (5) ‘dairy products (i.e. cheese and yogurt) consumed daily in low to moderate amounts’; (6) ‘fish and poultry consumed in low to moderate amounts’; (7) ‘0-4 eggs consumed weekly’; (8) ‘red meat consumed in low amounts’; (9) ‘wine consumed in low to moderate amounts, normally with meals’^(14)^Details: nutrient highlight – contribution of saturated fat to total energy needs is low, about < 8 % of energy; contribution of total fat to total energy needs vary widely across different Mediterranean regions, from < 25 % to >35 %^(14)^	Origin • Referred to the dietary pattern observed in the north-western Mediterranean region in 1960s • Popularised by Dr. Ancel Keys and the Seven Countries Study due to its evident association with heart health, cancer rates and other diet-related chronic diseases^(56,57)^ Evolution • Some earlier evidence included other lifestyle behaviors, such as eating with friends, but was lost in translation to the popular version^(14)^
EAT Lancet^(15)^ To simultaneously address chronic disease and climate change	Guidelines: ‘[A planetary health diet has] an optimal caloric intake and consist largely of a diversity of plant-based foods, low amounts of animal source foods, contain unsaturated rather than saturated fats, and limited amounts of refined grains, highly processed foods and added sugars’^(15)^ Details: dietary patterns by food groups and nutrients (2500 kcal/d; range of weight in grams or energetic intake/d) – whole grains (232 g or 811 kcal); tubers or starchy vegetables (0–100 g or 39 kcal); vegetable (200–600 g or 78 kcal); fruits (100–300 g or 126 kcal); dairy foods (0–500 g or 153 kcal); protein sources (0–386 or 726 kcal); added fats – unsaturated (20–80 g or 354 kcal) or saturated oils (0–11·8 g or 96 kcal); added sugars (0–31 g or 120 kcal)^(15)^	Origin • The world is lagged in meeting the United Nations Sustainable Development Goals (SDG) for human health and the Paris Agreement for environmental health^(14)^ • Scientific evidence suggests that a healthful diet is possible to address both human health and environmental health^(15)^ • Experts, recruited to develop a report that describes a EAT-Lancet reference diet, were from the following fields: human health, agriculture, political sciences and environmental sustainability^(15)^
NOVA^(37)^ To be able to decipher the healthfulness of a food item based on the level of food processing	Guideline: Avoid ultra-processed foods (NOVA group 4) and choose the least processed form of foods (NOVA groups 1–3 as feasible^(37)^. Details: classification Unprocessed or minimally processed foods (NOVA group: (1) describes foods that are consumed in its original form and the level of processing involves techniques like heat (i.e. steak) and grinding (i.e. flour for pasta) for food safety and palatability. Processed culinary ingredients (NOVA group; (2) are made of foods in group 1 but mostly used in small amounts for flavouring, seasoning and garnishing (i.e. butter, honey and corn starch). Processed foods (NOVA group; (3) added NOVA group 2 to group 1 plus preservatives sometimes to enhance flavour (i.e. pickles and cheese). Ultra-processed foods (UPF, NOVA group and (4) involve substantial amounts of some ingredients from NOVA group 2 (i.e. salt, sugar and fat) for the purpose of maximal flavouring, extended shelf-life and minimal production cost. Food products in NOVA group 4, such as carbonated beverages, packaged snacks and infant formulas, are often associated with increased risk of developing diet-related diseases: high in sugar, salt, fat and portion size and low in fresh or whole foods^(37)^.	Origin • Originated in Brazil^(58)^ • Began with a thesis to assess the relationship between industrial processing of food, nutrition and health^(37)^ • Mounting evidence suggests the association between food processing and disease^(37)^ • Varying levels of processed foods have a rapidly increasing presence in the food system^(37)^ • Big Food is driving the production of processed foods^(37)^ • ‘Big Food’ is inseparable from our everyday life^(37)^

## Cultural adaptability of popular and emerging dietary recommendations

We describe four dietary patterns or frameworks in terms of their cultural adaptability with detailed descriptions of each dietary recommendation and its origin and evolution provided in Table 1.

## Dietary approaches to stop hypertension

The DASH diet embodies a public health mission of addressing hypertension, implying its explicit emphasis on the absence of a specified disease and not the maintenance of well-being of a general population^(13)^.

Cultural adaptation of DASH is straightforward for food group recommendations but less so with nutrients^(13)^. Individuals lack data on nutrient composition of cultural foods that are not easily accessible to the public^(13)^, which requires substantial resources from both programme participants and implementers^(22–25)^. In addition to cultural adaptation of food- and nutrition-related materials, counseling sessions or food environment assessments were also included as part of the interventions. For example, two 10- to 12-week studies that each adapted DASH to Korean or Latin cultures included modifications on the unit of measure and examples of foods in each food group^(20,23)^ and multiple in-person and telephone sessions^(20,21)^. Another cultural adaptation of DASH was a 12-week randomised controlled pilot with African Americans by identifying traditional foods and dietary habits and including a food environment assessment of the participants’ neighbourhood^(24)^. These studies suggest DASH has been adapted across a number of cultures and populations and short-term health improvements. However, it is unclear whether DASH is feasible for individual- or community-level adaptation, and whether the treatment-oriented end-goal, i.e. reduce blood pressure, is appropriate for everyday use.

## Mediterranean diet

Mediterranean diet (MD) is similar to DASH in its emphasis on physical health but is also recognised as an ‘Intangible Cultural Heritage of Humanity’ by the United Nations Educational, Scientific and Cultural Organization^(6)^:

‘A set of skills, knowledge, rituals, symbols and traditions concerning…particularly the sharing and consumption of food. Eating together is the foundation of the cultural identity and continuity of communities…social exchange and communication…emphasizes values of hospitality, neighbourliness, intercultural dialogue and creativity, and a way of life guided by respect for diversity. It plays a vital role in…bringing together people of all ages, conditions and social classes…Markets also play a key role as spaces for cultivating and transmitting the Mediterranean diet during the daily practice of exchange, agreement and mutual respect.’^(6)^

It is noteworthy that while much of the United Nations Educational, Scientific and Cultural Organization description overlaps with multiple contextual factors which have been the focus of public health efforts in recent years – including social cohesion, community engagement and climate change^(22–24)^ – in the day-to-day understanding of this diet, these factors are largely absent. These upstream factors illustrate the important contexts in which MD is to be adapted, yet they are not translated into the popular version of MD, which focuses solely on food composition. Cultural adaptation of the current MD version means failure to account for the social connections with foods, which possibly compromises the benefits of following a MD in its entirety^(7)^.

MD has been culturally adapted to Latina/x/o and Black populations in the USA. The studies involving Latina/x/o specified surface-structure (e.g. language, food items) and, to varying degree, deep-structure adaptations (e.g. including family members) and also indicated good acceptability from Latina/x/o participants of the adapted MD plan^(21,25)^. The Heart Healthy Lenoir Project, which involved a majority of Black, low-income participants, culturally adapted by retaining Southeastern foods and focused on the quality of oil^(26)^. To our knowledge, no USA-based studies have adapted the MD for Asian subgroups despite their cardiometabolic risks being higher than whites and the USA general population^(11–15)^. To summarise, cultural adaptations of the MD that consider social and cultural elements to some degree seem to have been met with some success for diverse groups^(8,19)^. However, the broader question of whether it is even ‘appropriate’ to impose a cultural diet onto an entirely different culture persists.

## EAT-Lancet reference diet

Similar to MD and DASH, the EAT-Lancet Reference Diet (EAT) addresses physical health both directly through individual-level food selection and indirectly through system-level food production. EAT is relatively new and therefore little evidence of cultural adaptation exists, but current research points to the fact that components of EAT may serve as barriers to cultural adaptation. A global initiative, EAT claims that the dietary pattern is applicable to all food cultures, but its scientific basis, similar to DGA, is largely and narrowly drawn from literature based in North America^(15)^. We illustrate this through three observations. First, the suggestion of EAT to consume minimal/no animal source foods overlooks the European- and Asian-based studies demonstrating benefits in consuming amounts of animal source food higher than EAT’s recommendation^(27–31)^. Second, the food group recommendations in EAT undercut the ‘plant-based’ diets that some cultures, such as those in Southeast Asia, have already followed throughout the millennia^(32,33)^. Lastly, the emphasis in EAT to shift to plant-based diet may create financial burden for racial/ethnic minority populations, who disproportionately experience food insecurity in an environment where plant-based foods remain more costly compared to meat or processed foods in terms of energy density^(34,35)^. To summarise, EAT’s global reach and its considerations for environmental health through dietary change demonstrate a shift away from the focus of only physical health. Moreover, its narrow scientific evidence makes adherence to EAT less feasible for diverse communities, in some cases may be inadvisable, and takes a primarily Eurocentric/meat-based approach.

## NOVA classification system

In contrast to DGA, MD, DASH and EAT that make recommendations for food groups based on their nutrient composition and density, the NOVA Classification System (NOVA) provides a framework for classifying single food items according to the level of processing. The origin of NOVA is similar, however, to the other DR in that it was developed out of concern for ultra-processed foods and their detrimental impacts on physical health^(36)^. NOVA is by design considerate of cultural diversity by encouraging the shift of food choices to less processed foods to opting for regional/local ingredients that are less likely to require a higher level of processing for food preservation and/or storage^(37)^. Moving away from ultra-processed foods means preserving food culture at the local level on the global landscape. No studies to date have engaged in cultural adaptation of NOVA in the USA, except for a study that suggests nutrition education involving NOVA to be appropriate for a racially/ethnically diverse group of college students^(36)^. In summary, despite a lack of evidence thus far, NOVA with its simple to follow message that takes into consideration sociocultural factors may be a viable option as a basis for nudging individuals towards better nutrition across multiple diverse groups.

## Discussion

By 2045, the USA will become a ‘majority minority’ community^(38)^, but DR do not serve all Americans equitably. They rely on evidence derived from the general USA population and/or a Eurocentric perspective, with regards to foods consumed, food choices, affordability and underlying nutrition profile. The importance of eating is reduced from a sociocultural significance to a carrier of nutrients for physical health.

The DR described in this commentary differ in their origin and evolution but are similar in their reductionist emphasis on physical health. The cultural adaptation interventions we examined here are limited by scarcity of resources and, for the most part, limited to adaptation at the surface level^(38)^, which is likely due to the fact that social science literature that pertains to food and culture^(9,39,40)^ is largely absent in the science base of the DR. Existing efforts through Oldways^(41)^ and Med Diet 4·0 framework^(42)^ that go beyond the ‘physical health’ framing to embody the social and cultural aspects of MD are promising, but more difficult, time-consuming and infrastructure change efforts will be needed to operationalise such changes^(43)^.

Despite the shortfalls, we are inspired by some elements of these DR and suggest four key aspects for consideration when developing DR centered on cultural orientation: (1) to address nutrition and health in the context of food and cultural studies, practices and history; (2) to actively engage racial/ethnic minorities and immigrants to explore their preferences and traditions they wish to preserve and document them to build the evidence base; (3) to distinguish dietary patterns that are for disease treatment (i.e. DASH for hypertension) from those that are for maintaining health (DGA for generally healthy Americans) or for preventing certain groups of disease (i.e. NOVA for metabolic disorders) and promote them accordingly and (4) to focus on strengths not on deficits of the racial/ethnic minority foods and culture of eating.

Adherence to the current DR may support physical health but may compromise social and emotional health^(44)^ and, in some cases, ethnic identity and well-being^(45)^. The demographic shift towards a more diverse population means our evidence base for DR needs to be reflective of the racial and ethnic diversity and associated diversity of food cultures. Given the rapidly growing interest in precision nutrition^(46,47)^ and food as medicine^(48,49)^ that are oriented to physical health, it is urgent that policymakers and researchers think about the values we want to preserve for future generations and the role of culture in nutrition and health without perpetuating health inequities in the USA.
